# Two issues should be noted when designing a clinical trial to evaluate BCG effects on COVID-19

**DOI:** 10.3389/fimmu.2023.1207212

**Published:** 2023-05-23

**Authors:** Wenping Gong, Yinping Liu, Yong Xue, Li Zhuang

**Affiliations:** ^1^Beijing Key Laboratory of New Techniques of Tuberculosis Diagnosis and Treatment, Senior Department of Tuberculosis, The 8th Medical Center of PLA General Hospital, Beijing, China; ^2^Hebei North University, Zhangjiakou, Hebei, China

**Keywords:** Bacillus Calmette-Guérin (BCG), tuberculosis (TB), COVID-19, clinical trial, latent tuberculosis infection (LTBI)

## Introduction

Bacillus Calmette-Guérin (BCG) is the only licensed vaccine for tuberculosis (TB) prevention (1). A systematic review evaluated the effects on non-specific and all-cause mortality in children under 5 vaccinated with BCG, and the results showed that BCG vaccination was associated with reduced all-cause mortality, with an average relative risk of 0.70 in the five clinical trials and 0.47 in the nine high-bias observational studies included in the analysis (2). Another study conducted in northern Sweden found similar results that BCG vaccination was associated with a reduction in non-TB infectious deaths (RR = 0.75 (0.58-0.97), especially respiratory infections (3). Similarly, a case-control study in southeastern Brazil found that BCG reduced the risk of death from pneumonia in children under 1 year of age by 50% (4). These studies demonstrated that BCG vaccination can induce non-specific protective effects against non-mycobacterial infections. These non-specific protective effects may be related to three immunological mechanisms: trained immunity, heterologous immunity, and anti-inflammatory effect (5).

BCG-induced trained immunity has attracted the most interest of the three immune mechanisms described above. BCG-induced trained immunity involves the reprogramming of innate immune cells through epigenetic reprogramming (such as DNA modifications, noncoding RNAs, histone modifications, and chromatin remodeling) (6, 7), metabolic reprogramming (such as glycolysis, oxidative phosphorylation, and glutamine catabolism pathways) (8, 9), and long-term protection mediated by hematopoietic stem cells (10), resulting in enhanced responsiveness and protection against a range of infections (11). Therefore, BCG has been widely used in cancer therapy, infectious disease prevention, autoimmune disease treatment, and other diseases such as Alzheimer’s disease and Parkinson’s disease (11–13).

Based on the above facts, a hypothesis has been proposed in recent years: BCG-induced trained immunity can reduce the incidence, hospitalization, severe disease, and mortality of COVID-19 (14). Therefore, in the early stage of the COVID-19 pandemic, researchers conducted a large number of ecological, epidemiological, and statistical analyses on the potential of BCG to reduce COVID-19 morbidity and mortality, based on the incidence and mortality reported by the World Health Organization (WHO) and individual countries, supplemented by BCG vaccination rates in individual countries (5). However, the results of these observational studies are often highly heterogeneous due to many confounding factors. To address this challenge, researchers have set out to test this hypothesis in clinical trials with relatively high levels of evidence. Over 56 clinical trials were conducted in the Netherlands, India, Brazil, Denmark, Germany, the USA, and Greece to assess the effect of BCG vaccination on reducing the number of infections, days away from work, hospital admissions, morbidity, and mortality (15). To date, ten of these trials have published their results, including NCT04659941 (16), NCT04435379 (17), NCT04414267 (18), NCT04379336 (19), NCT04328441 (20, 21), RBR-4kjqtg (22), NCT04417335 (23), NCT04648800 (24), and CTRI/2020/07/026668 (25) (**Table 1**). However, the results of these clinical trials remain heterogeneous, making it impossible to draw any firm conclusions from them. Thus, we make the following recommendations for the design of clinical trials to evaluate the protective effect of BCG on COVID-19, which may help to address these heterogeneities.

**Table 1:** Randomized controlled trials (RCTs) on evaluating BCG vaccination effectiveness against COVID-19.

NCT number (reference)	Study Design	Participants characteristics		Intervention		Follow-up	Results	TB status
		Mean Age (Year)	Total number (Male, %)	BCG stain and dosage	Placebo			
NCT04435379 (17)	Phase 3 mRCT	67.3	2025 (52.9)	VPM1002, 2–8 × 10^5^ CFUs	Saline solution	240 days	VPM1002 vaccination is safe and seems to protect elderly individuals from severe respiratory disease.	Individuals with ATB or LTBI were excluded
NCT04414267 (18)	Phase 3 sRCT	69	301 (67.8)	0.1 mL BCG Moscow	Saline solution	6 months	BCG vaccination can offer some protection against COVID-19 among individuals over 50 years old with underlying health conditions.	Individuals with ATB or LTBI were excluded
RBR-4kjqtg (22)	Phase 2 sRCT	43	131 (23.7)	0.1 mL BCG Moscow	–	180 days	A second BCG Moscow vaccination was linked to a reduced rate of COVID-19 infections, although the findings were not statistically significant.	Individuals with ATB or LTBI were excluded
NCT04417335 (23)	Phase 3 sRCT	67	2014 (52.5)	0.1 mL BCG Danish strain	Saline solution	12 months	BCG vaccination did not impact the occurrence of SARS-CoV-2 infection among older adult volunteers. However, it did enhance the cytokine responses produced by both influenza and SARS-CoV-2, as well as lead to stronger antibody titers following COVID-19 infection.	Individuals with ATB or LTBI were excluded
NCT04328441 (20)	Phase 3 mRCT	42	1511 (25.7)	0.1 mL BCG Danish strain	Saline solution	26 weeks	BCG vaccination offers some defense against potential COVID-19 infection in patients over 50 years old who have underlying health conditions.	Individuals with ATB or LTBI were excluded
NCT04328441 (21)	Phase 3 mRCT		1309 (25.6)	0.1 mL BCG Danish strain 1331	Saline solution	12 months	BCG vaccination did not reduce the incidence of SARS-CoV-2 infection in HCWs, nor did it reduce the duration or severity of infection, but it may enhance antibody production during SARS-CoV-2 infection.	Individuals with ATB or LTBI were excluded
NCT04379336 (19)	Phase 3 mRCT	39	1000 (29.6)	0.1 mL BCG Danish strain	Saline solution	52 weeks	BCG did not protect HCWs from SARS-CoV-2 infection or related severe COVID-19 disease and hospitalization.	Individuals with ATB were excluded
NCT04648800 (24)	Phase 3 mRCT	45	342 (19.3)	0.1 mL BCG Moreau strain	Saline solution	3 months	There was no meaningful association found between the frequency of suspected COVID-19 incidents and BCG-10 vaccination, tuberculin test results, or the number of scars.	Individuals with a TB history were excluded
NCT04659941 (16)	Phase 2b mRCT	Groups	264 (20.4)	0.1 mL BCG Moreau and Moscow strains	Sterile 0.9% NaCl	180 days	BCG did not demonstrate a protective hazard ratio against COVID-19.	Individuals with ATB or LTBI were included
CTRI/2020/07/026668 (25)	Phase 3 mRCT	43	495 (52.1)	0.1 mL BCG, 0.2 and 0.8 million CFUs	0.1 ml of normal saline	6 months	BCG vaccination did not significantly reduce the incidence of PCR-positive COVID-19 infection but did significantly reduce the incidence of clinically diagnosed COVID-19 infection in high-risk population.	Individuals with ATB or LTBI were not determined

CFU, colony forming unit; HCWs, health care workers; NCT, Clinical trials registration number; VPM1002 vaccine is a genetically modified BCG; sRCT, single center randomized controlled trial; mRCT, multicenter randomized controlled trial.

## Participants with ATB and LTBI should be excluded from the clinical trial

The BCG-induced trained immunity can be interfered by the existence of immune responses induced by the *Mycobacterium tuberculosis*, which should be indirectly detected by tuberculin skin test (TST) or *M. tuberculosis* antigen-specific interferon-gamma release assays (IGRAs) (18). Early in the COVID-19 pandemic, epidemiologic data from Europe suggested a lower prevalence of COVID-19 in individuals with a higher prevalence of latent tuberculosis infection (LTBI) [r(20): - 0.5511 ~ - 0.6338; *p* < 0.001] (26). Similarly, a retrospective study from Turkey investigated the association between TST induration diameter and the clinical course of COVID-19 and showed that TST positivity was higher in patients with milder COVID-19 (*P*<0.001) and a large induration diameter (*P*<0.001) (27). Another study evaluated the severity of disease in COVID-19 patients in relation to LTBI infection rates and its correlation with laboratory parameters and clinical outcomes and found that individuals with LTBI who developed COVID-19 had significantly less severe disease, accompanied by higher lymphocyte and monocyte counts (28). These findings are interesting and suggest a potential protective effect of LTBI against COVID-19. However, it is important to note that correlation does not necessarily equal causation and these findings need to be interpreted with caution. It is possible that other factors, such as age, comorbidities, and socioeconomic status, may confound these findings. Regardless, further research is needed to better understand the relationship between LTBI and COVID-19 outcomes. If a protective effect of LTBI can be confirmed, including participants with LTBI may confound the evaluation of BCG efficacy in protecting against COVID-19.

We investigated ten randomized controlled trials (RCTs) published in the last two years (**Table 1**), including six clinical trials indeed excluded participants with ATB or LTBI (17, 18, 20, 22, 23), two clinical trials excluded participants with ATB or a history of ATB (19, 24), one clinical trial included participants with ATB or LTBI (16), and one clinical trial didn’t report the status of ATB and LTBI (25). Interestingly, we found that four trials that excluded participants with ATB and LTBI supported a possible protective effect of BCG against COVID-19 (17, 18, 22, 23), and two studies showed that BCG vaccination did not reduce absenteeism, incidence, hospitalization, or associated severe COVID-19 disease among healthcare workers (HCWs) (20, 21). In contrast, two clinical trials that excluded only participants with ATB or a history of ATB but not LTBI suggested that BCG vaccination did not reduce the incidence, hospitalization, or associated severe COVID-19 disease in HCWs (19, 24). In a recent study, “The effect of BCG vaccination on infection and antibody levels against SARS-CoV-2-The results of ProBCG: a multicenter randomized clinical trial in Brazil”, published in the International Journal of Infectious Diseases, the authors found that BCG did not show a protective hazard ratio against COVID-19 (16), consistent with two clinical trials only excluded participants with ATB or an ATB history (19, 24). Furthermore, we found that the participants with ATB or LTBI were included in this study. The authors stated that the original inclusion and exclusion criteria for this study were HCWs 18 years of age or older who agreed to provide written informed consent, excluding pregnant women and those who had received a COVID-19-specific vaccine. Therefore, this clinical trial failed to exclude individuals with ATB and LTBI, making it impossible to distinguish the trained immunity induced by *M. tuberculosis* infection from those induced by BCG. In December 2022, Sanjeev Sinha and colleagues published a phase III multicenter RCT in *Infectious Diseases and Therapy* to evaluate the protective efficacy of BCG against COVID-19 in high-risk populations (25). The results showed that BCG vaccination could not significantly reduce the incidence of PCR-positive COVID-19 in high-risk populations but could reduce the incidence of clinically diagnosed COVID-19. A major limitation of the study was that it did not explicitly report the ATB and LTBI status of the enrolled participants.

Based on the expanding evidence supporting the association between LTBI and COVID-19 outcomes, we propose that randomized controlled trials (RCTs) aimed at assessing the protective efficacy of BCG vaccination against COVID-19 should exclude participants with ATB or LTBI, as determined by either the TST or IGRAs, during the trial design stage. This exclusion will enhance precision and accuracy of the trial results by controlling for potential confounders and ensuring that the observed association, if any, between BCG vaccination and COVID-19 efficacy is attributable to the vaccination alone. This recommendation should be further evaluated and implemented by the scientific and medical communities to optimize the design of upcoming RCTs.

## One clinical trial would ideally use one BCG strain

Since its initial development in 1921, the Bacillus Calmette-Guérin (BCG) vaccine has undergone various modifications, resulting in several strains, including early strains such as BCG Russia, Japan, Moreau, and Sweden, and later strains such as BCG Denmark, BCG Tice, BCG Sweden, BCG Frappier, BCG Pasteur, BCG Prague, BCG Glaxo, and BCG Connaught (29). These various strains exhibit differences in immunogenicity, protective efficiency, and adverse effects. A comparative study utilizing the guinea pig model of pulmonary tuberculosis, evaluated the protection efficacy of six commonly used BCG strains, including BCG Japanese, BCG Danish, BCG Glaxo, BCG Connaught, BCG Pasteur, and BCG Tice, showing that BCG Tice had the best protection efficiency, while BCG Glaxo had relatively poor protection (30). Interestingly, an additional randomized trial conducted in Hong Kong involving 303,092 neonates showed that the incidence of TB after vaccination with BCG-Glaxo was 1.8 times higher than that following vaccination with BCG-Pasteur (31). A cohort study in Kazakhstan found that BCG Japan, BCG Serbia, and BCG Russia provided 69%, 43%, and 22% protection against clinically diagnosed TB and 92%, 82%, and 51% protection against culture-confirmed TB, respectively (32).

Furthermore, studies indicate that BCG strains differ not only in the prevention of TB, but also in the therapeutic effects of BCG on non-muscle-invasive bladder cancers (33, 34). A separate study involving Brazilian infants revealed differing cytokine expression profiles elicited by BCG Moro and BCG Russian strains, with BCG Moro inducing higher levels of cytokines such as IL-2, IL-4, and IL-10, compared to BCG Russian (35).

These findings highlight the importance of considering the type of BCG strains used in evaluating its protective efficacy against COVID-19 (36). Some BCG strains may act as a confounding factor, thus affecting the outcomes of clinical trials. Among the four BCG strains used in previously published clinical trials (**Table 1**), BCG Moscow, BCG Danish, BCG Moreau strains, and VPM1002 (a genetically modified BCG) strain were evaluated. Additionally, nine clinical trials with participant numbers ranging from 131 to 2025 assessed the efficacy of specific strains of BCG against COVID-19 (17–25). One study with a sample size of only 264 participants evaluated the protective efficacy of BCG Moreau and Moscow strains against COVID-19 (16). Although this study did not reach its originally calculated sample size of 376 individuals for each group, the findings provide valuable insights, even when considering the further subdivision of the BCG group into the BCG Moreau and BCG Moscow subgroups, which reduced the sample size in each group. Future clinical trials should consider these findings when designing trials to evaluate the protective efficacy of different BCG strains against COVID-19.

## Conclusions

In summary, to ensure the accuracy of RCTs evaluating the protective effect of BCG against COVID-19, we recommend excluding individuals with a history of TB, including ATB and LTBI. Furthermore, the usage of two or more BCG strains in a clinical trial should be avoided unless the sample size of each subgroup is large enough to compensate for potential confounding factors. These guidelines can help prevent bias and enhance the validity and generalizability of clinical trial results, ultimately contributing to the development of effective strategies for COVID-19 prevention and control.

## Author contributions

Conceptualization: WG; Data collection and data analysis: WG, YL, YX, and LZ; Funding acquisition: WG; Software: YL, YX, and LZ; Writing - original draft: WG; Writing - review and editing: WPG, YPL, YX, and LZ. All authors contributed to the article and approved the submitted version.
